# Changes in breast cancer incidence and surgical treatment in Baden–Württemberg (Germany) during the COVID-19 pandemic

**DOI:** 10.1038/s41598-024-75084-y

**Published:** 2024-10-22

**Authors:** Lina Jansen, Silke Hermann, Susanne Bergbold, Volker Arndt

**Affiliations:** Epidemiological Cancer Registry Baden-Württemberg, German Cancer Research Center (DKFZ), Im Neuenheimer Feld 581, 69120 Heidelberg, Germany

**Keywords:** Breast cancer, COVID-19, Incidence, Surgery, Mastectomy, Germany, Breast cancer, Cancer epidemiology, Cancer therapy

## Abstract

The COVID-19 pandemic affected the diagnostics and treatment of breast cancer. Numerous studies reported an early decline in breast cancer (BC) incidence during the COVID-19 pandemic. Less evidence is available on changes in medical care. Reports from individual patients have provided anecdotal evidence for a shift from breast-conserving surgery to mastectomy to reduce the number of visits to radiation units during the pandemic. This study aimed to explore changes in BC incidence and surgical treatment in the south of Germany. Using data from the Baden-Württemberg Cancer Registry, the age-standardized incidence of BC (ICD-10 C50 and D05) (women) in 2018–2021 was investigated overall and by age and stage using standardized incidence ratios. Among pre-operative stage I/IIA BC patients, differences in the time to surgery and type of surgery were investigated using negative binomial and logistic regression models. The incidence of invasive BC decreased significantly from 170.9 per 100,000 women in 2018/2019 to 159.7 in 2020 and increased to 169.2 in 2021. This decrease resulted from a lower incidence around April 2020 and was also observed for non-invasive BC. In 2021, incidence of invasive BC was still decreased by 8% in women aged 80 + years. Surgical treatment was analyzed in 22,708 BC patients with a pre-operative stage ≤ IIA. The median time to surgery was 33 days in 2018/2019, 32 days in 2020 and 36 days in 2021. The proportion of mastectomies increased from 16.1% in 2018/2019 to 17.1% in 2020 and 17.3% in 2021 (adjusted odds ratio and 95% confidence interval (2021 vs. 2018/2019): 1.13 (1.03–1.24)). The adjusted increase was strongest for patients aged 50–59 years (1.34 (1.09–1.64)) and those with high-grade tumors (1.27 (1.07–1.51)). While the early return to pre-pandemic age-standardized BC incidence rates is promising, missed cases have not been caught up until 2021. Furthermore, the decreased incidence in elderly women in 2021 warrants further attention. In early-stage BC, a slightly greater rate of mastectomies was observed, although such a change was not recommended. This result underlines the importance of good communication of adapted treatment guidelines in such exceptional circumstances.

## Introduction

Breast cancer (BC) is the most common cancer in women worldwide and accounts for 23.8% of all annual cancer cases^1^. In Germany, approximately 70,550 new invasive BCs and 6,000 new non-invasive BCs are diagnosed annually^2^. The introduction of organized mammography screening in Germany in 2005–2009 led to a reduction in the incidence of late-stage BC in screening age groups (50–69 years)^3^. In 2019/20, most patients were diagnosed with stage I (40%) or II disease (41%)^2^. BC treatment depends strongly on stage and molecular subtype^4^. For patients with early-stage BC (stage I to IIA), breast-conserving surgery (BCS) followed by radiotherapy (accompanied by endocrine therapy, depending on hormone receptor status) is currently the standard treatment^4,5^.

In March 2020, the World Health Organization declared the coronavirus disease 2019 (COVID-19) outbreak a pandemic. Consequently, many countries suspended their mammography screening programs for some months. This intervention led to a decrease in the incidence of BC^15^. In Germany, the first lockdown started at the end of March and lasted seven weeks. In April 2020, the mammography screening program was paused for four to six weeks^6^. Due to the transformation of medical resources from surgery to emergency care, the treatment of cancer patients might have been delayed. Adapted treatment recommendations have rapidly developed^7^. With respect to surgery, it was suggested that endocrine therapy be administered pre-surgically to delay surgery. Surgery for non-invasive BC (except for extended high-risk ductal carcinoma in situ) and for breast reconstruction, was given low priority. Anecdotal evidence from patient reports at the Cancer Information Service in Germany suggested that there may have been a preference for mastectomy to avoid adjuvant radiotherapy during the pandemic.

The aim of this study was to investigate changes in BC incidence and in surgical treatment of early-stage BC in the federal state of Baden–Württemberg (Germany) in the 2020/2021 pandemic years compared to the pre-pandemic years 2018/2019. Following the adapted treatment guidelines and the anecdotal evidence, changes in time to surgery and mastectomy rates were investigated in detail.

## Methods

### Study population

The study is based on data from the Baden–Württemberg Cancer Registry. The cancer registry collects information on all cancer cases who live in or were treated in Baden–Württemberg. The registry covers a population of 11.28 million people (end of 2022) in the south-west of Germany. Reporting of cancer cases is mandatory for all physicians and health care providers involved in the diagnosis or treatment of cancer. Notification events are diagnosis, pathology report, specific cancer therapy, disease progression or unremarkable follow-up or death, and optional tumor conference. In Germany, a uniform national and legally binding oncology data set defines the basis for reporting. In Baden–Württemberg, mandatory reporting was introduced in 2009–2011.

Women living in Baden–Württemberg with a primary invasive (International Classification of Diseases (ICD)-10: C50) or non-invasive (D05) BC diagnosis in 2018–2021 were included in the incidence analyses. If the month of diagnosis was not reported to the cancer registry or was marked as “estimated” by the notifying physician/pathologist, the case was excluded (0.6%).

**Fig. 1:**
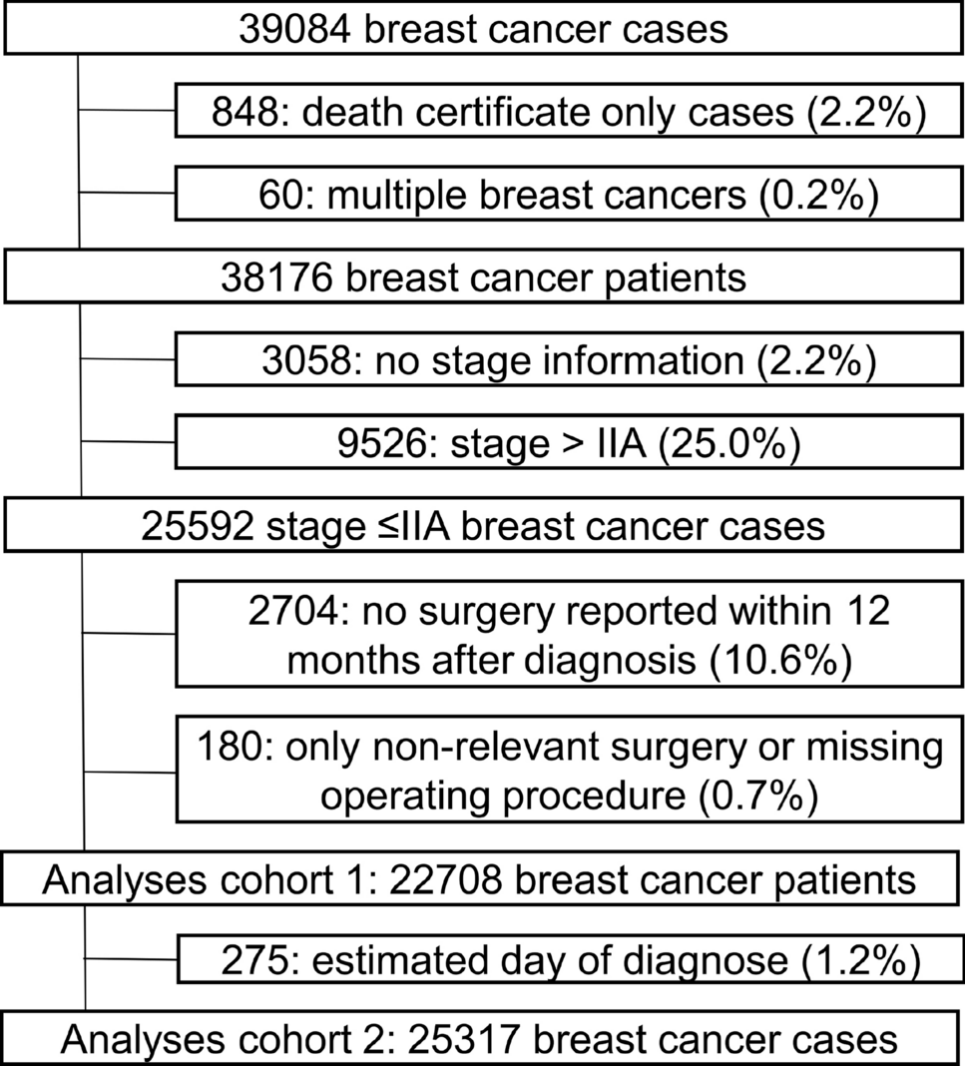
Selection of the cohort for type of (1) and time to (2) surgery analyses.

In analyses of time to and type of surgery, the cohort was restricted to early-stage BC patients (Union for International Cancer Control (UICC) stage lower or equal to IIA prior to surgery) who underwent surgical treatment (BCS or mastectomy). The restriction was made to define a homogeneous group of patients for whom BCS without neoadjuvant treatment was the standard of care prior to the pandemic. Patients notified by a death certificate only (DCO) and patients without notification of BCS or mastectomy were excluded. In analyses on time to surgery, patients with an estimated day of diagnosis or surgery were also excluded. Figure 1 illustrates the selection process.

### General population

For incidence analyses, data on the female mid-population in Baden–Württemberg by calendar year and age were obtained from the statistical offices of the German states^8^. Monthly population data were not available, and thus, a constant population was assumed for the whole year.

### Classification of variables

For incidence analyses, the UICC stage was derived, including all information collected within three months after diagnosis, according to the German Cancer Registration manual^9,10^. For surgical treatment analyses, only staging information before surgery was considered. If no staging information before surgery was available, the above-described stage was used as an approximation (n = 2,150, 5.6%). If metastatic information was missing, M0 status was assumed. To subdivide the 2020/2021 pandemic into phases based on the pandemic situation, published cut-offs were used to classify the following phases: first sporadic cases (sporadic), 1st wave of COVID-19 (W1), 2nd wave of COVID-19 (W2), 3rd wave with variant of concern alpha (W3), 4th wave (delta, W4) segmented in summer (a) and autumn/winter (b), and summer plateaus (S)^11^. The years 2018 and 2019 served as reference years.

The histological codes (ICD-O-3) were categorized as ductal carcinoma of no special type, lobular carcinoma, ductal and lobular carcinoma, other specific histology, or unspecified. The codes used are provided in Table 1.

**Table 1:** Classification of histological groups.

Histological group	ICD-O-3 morphology codes
Ductal	8500
Lobular	8520, 8524
Ductal & Lobular	8522
Unspecified	8000, 8001, 8010
Other, specified	Other codes than those listed above

The estrogen receptor (ER), progesterone receptor (PR), and human epidermal growth factor receptor 2 (HER2) receptor statuses were classified as positive or negative. Hormone receptor status was classified as positive (HR +) in patients who were ER + or PR + .

Surgery was classified using the German procedure classification (Operationen- und Prozedurenschlüssel – OPS). BCS was defined by the codes “5–870” and “5–871”. The codes “5–872” to “5–877” were defined as “mastectomy”. The first mastectomy or BCS within one year after diagnosis was defined as the surgery of interest.

To support the interpretation of the main analyses, data on neoadjuvant therapy and adjuvant radiotherapy were extracted from the cancer registry database. These variables are collected by the registry but standardized aggregation of individual treatment reports into a best-of dataset has finally not yet been implemented. Furthermore, registration is likely to be incomplete, especially for hormone therapy, and validation of these treatment factors has not yet been conducted. Neoadjuvant therapy was defined as start of therapy after diagnosis but prior to surgery. Neoadjuvant endocrine therapy was defined as neoadjuvant therapy indicated as endocrine therapy. We defined adjuvant radiotherapy as the start of radiotherapy from date of surgery up to 6 months after surgery.

### Statistical analyses: incidence analyses

The incidence was age-standardized to the population of Baden–Württemberg in 2021 using 5-year age groups up to 85 + years and reported as cases per 100,000 person-years. Age-specific incidence estimates were standardized within age groups following the same approach. The incidences in 2020 and 2021 were statistically compared to those in the reference period (2018/2019) by standardized incidence ratios. Stratified analyses were conducted for invasive and non-invasive BC and by age and stage.

### Statistical analyses: time to surgery and type of surgery

Characteristics of BC patients were described by calendar period. Distributions were compared using Chi-square tests for categorical and Welch’s test for continuous variables.

As neoadjuvant therapy increases the time to surgery, the proportion of patients who received any neoadjuvant therapy was reported overall and by age, grade, pre-surgery stage, HR and HER2 status. In addition, the proportion of HR+ patients who received neoadjuvant endocrine therapy was computed.

Time to surgery was reported as median and mean by calendar period and month overall and by calendar period and age, grade, stage before surgery, HR and HER2 status, and neoadjuvant therapy. Differences were statistically tested using a negative binomial regression model with number of days between diagnosis and surgery as dependent and the above-described factors (with and without neoadjuvant treatment) as independent or stratification variables. Incidence rate ratios (IRR) and 95% confidence intervals were extracted. In these models, the IRR shows the factor change in time to surgery in days that is associated with an increase in the independent variable by one, while holding all other variables in the model constant.

The proportion of patients who underwent mastectomy was calculated for the same subgroups as in the time-to-surgery analyses. Odd of mastectomy during the pandemic and reference years were compared using a logistic regression model with type of surgery (reference: BCS) as the dependent variable and the above listed factors as independent or stratification variables.

As changes in mastectomy rates can lead to changes in the utilization of adjuvant radiotherapy, this factor was additionally investigated. The proportion of patients receiving adjuvant radiotherapy were calculated overall, by grade and by type of surgery. Odds ratios were computed using the same model as for the odds of mastectomy with adjuvant radiotherapy (reference = no) as dependent variable.

### Statistical analyses: general aspects

The number of patients with missing information is shown in the tables. Patients with missing information for relevant variables were excluded from the analyses. A *p* value < 0.05 was considered to indicate statistical significance. No multiple comparison corrections were conducted.

## Results

### Incidence

From 2018 to 2021, 39,084 invasive and 2,541 non-invasive BCs were reported to the Baden–Württemberg Cancer Registry. Of the invasive cases, 848 (2.2%) were known only by death certificate, and 110 (0.3%) had an estimated month of diagnosis, with comparable rates across the years.

**Fig. 2:**
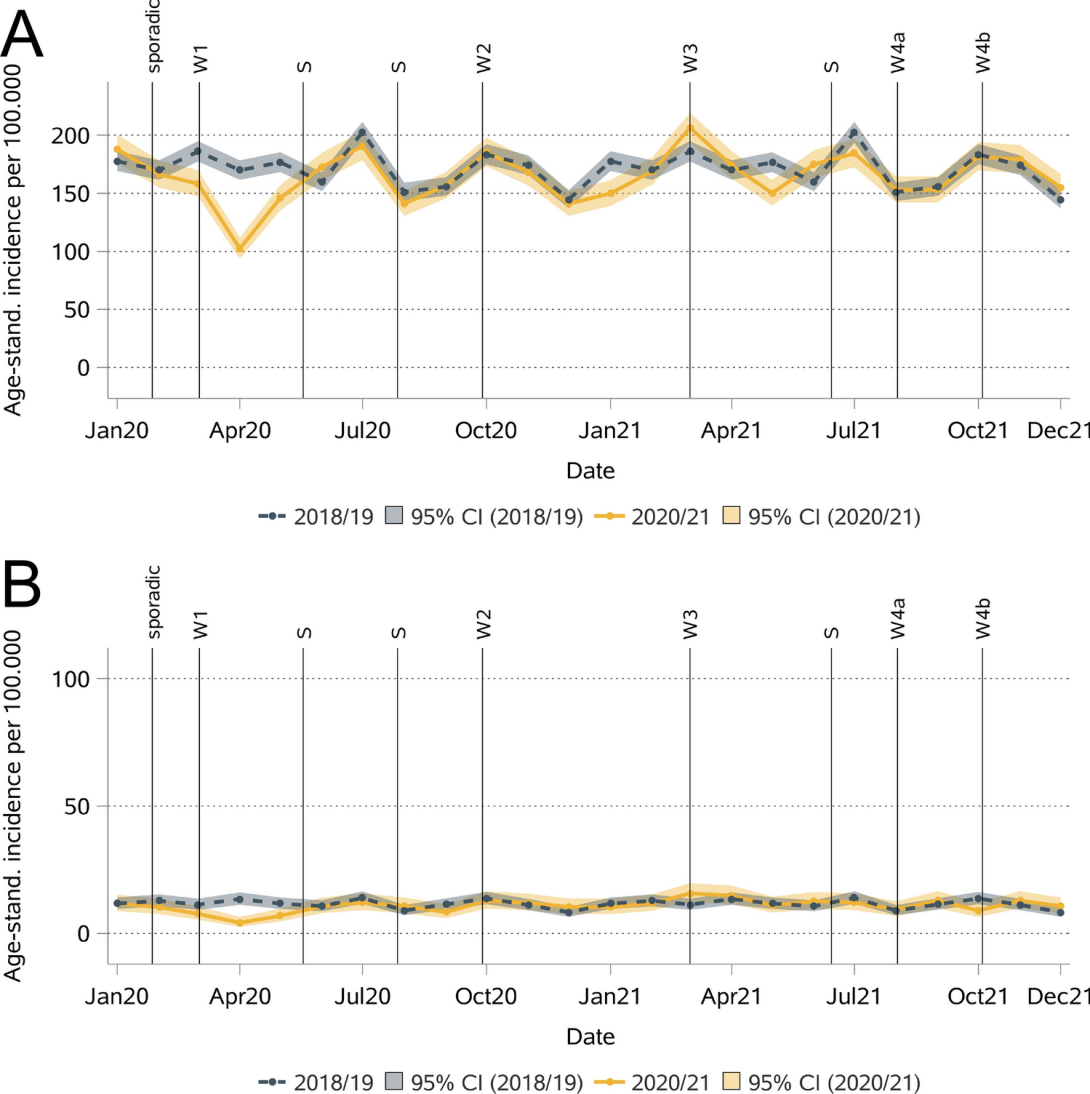
Monthly age-standardized incidences for invasive (**A**) and non-invasive (**B**) breast cancer.

The incidence of invasive BC decreased strongly during the first COVID-19 wave in 2020, but by summer 2020, it was already comparable to that in the reference years (Fig. 2). The largest decrease (-40%) was observed in April 2020, with an incidence of 102.5 (per 100,000 persons, 95% confidence interval (CI): 93.7–111.9) compared to 169.8 (161.6–178.3) in 2018/2019. Overall, the age-standardized incidence of invasive BC was 7% lower in 2020 than in 2018/2019 (Table 2). In 2021, it was comparable to that in 2018/2019. The patterns were similar for non-invasive BC with an incidence of 4.2 (2.6–6.5) per 100,000 women in April 2020 compared to 13.5 (11.2–16.1) in 2018/2019 (-69%). Over the entire year, the incidence decreased from 11.6 in 2018/2019 to 9.8 in 2020 and increased thereafter to 11.9 in 2021.

**Table 2:** Age-standardized incidence for invasive and non-invasive breast cancer during the pandemic and reference years.

Site	Subgroup^a^	Incidence (95% CI) in the calendar year^b^			Comparison to 2018/2019	
ICD-10		2018/2019	2020	2021	IRR 2020 (95% CI)	IRR 2021 (95% CI)
C50	All	170.9 (168.5–173.3)	159.7 (156.5–163.0)	169.2 (165.9–172.6)	**0.93 (0.91–0.96)**	0.99 (0.97–1.01)
	Age < 50	48.7 (47.0–50.4)	46.9 (44.5–49.4)	50.2 (47.7–52.7)	0.96 (0.90–1.02)	1.03 (0.97–1.09)
	Age 50–59	256.6 (249.1–264.2)	244.6 (234.3–255.2)	258.9 (248.3–269.9)	0.95 (0.91–1.00)	1.01 (0.96–1.06)
	Age 60–69	355.3 (345.3–365.6)	310.5 (297.5–323.9)	351.2 (337.5–365.3)	**0.87 (0.83–0.92)**	0.99 (0.94–1.04)
	Age 70–79	378.1 (366.2–390.2)	368.0 (351.5–385.1)	375.7 (358.9–393.1)	0.97 (0.92–1.03)	0.99 (0.94–1.05)
	Age 80 +	401.7 (388.2–415.6)	374.0 (356.2–392.4)	369.7 (352.3–387.7)	**0.93 (0.88–0.99)**	**0.92 (0.87–0.98)**
	Stage I	59.3 (57.9–60.7)	54.7 (52.8–56.7)	59.3 (57.3–61.3)	**0.92 (0.88–0.96)**	1.00 (0.96–1.04)
	Stage II	59.9 (58.5–61.3)	55.5 (53.6–57.5)	57.2 (55.3–59.2)	**0.93 (0.89–0.97)**	**0.96 (0.92–1.00)**
	Stage III	15.9 (15.2–16.6)	14.0 (13.1–15.0)	14.4 (13.4–15.4)	**0.88 (0.81–0.95)**	**0.91 (0.84–0.99)**
	Stage IV	11.6 (11.0–12.2)	11.4 (10.6–12.3)	11.7 (10.8–12.6)	0.99 (0.90–1.08)	1.01 (0.92–1.11)
	Stage X	20.7 (19.9–21.6)	21.4 (20.2–22.6)	22.5 (21.3–23.8)	1.03 (0.96–1.11)	**1.09 (1.02–1.17)**
D05	All	11.6 (11.0–12.3)	9.8 (9.0–10.7)	11.9 (11.0–12.8)	**0.85 (0.77–0.93)**	1.03 (0.94–1.13)
	Age < 50	2.6 (2.2–3)	2.2 (1.7–2.8)	3.0 (2.4–3.7)	0.88 (0.66–1.16)	1.16 (0.90–1.50)
	Age 50–59	29.1 (26.6–31.7)	24.4 (21.2–27.9)	30.6 (27.0–34.5)	**0.84 (0.72–0.99)**	1.05 (0.91–1.22)
	Age 60–69	30.6 (27.7–33.7)	26.1 (22.4–30.2)	30.8 (26.9–35.2)	0.85 (0.71–1.01)	1.00 (0.85–1.18)
	Age 70–79	15.6 (13.2–18.2)	12.9 (9.9–16.5)	12.5 (9.6–16.0)	0.83 (0.62–1.11)	0.80 (0.60–1.08)
	Age 80 +	7.3 (5.6–9.4)	5.9 (3.8–8.7)	8.8 (6.3–12.0)	0.81 (0.51–1.29)	1.21 (0.81–1.80)

IRR = incidence rate ratio, CI = confidence interval; significant values are printed in bold; ^a^ Subgroups by age at diagnosis and sex. In stage-specific analyses, 41 (0.1%) patients with tumors that were not classifiable by the UICC according to their histology and 848 patients whose death certificate was available (2.2%) were excluded. Patients without stage information were classified as stage X. ^b^ Incidence per 100.000 women.

**Fig. 3:**
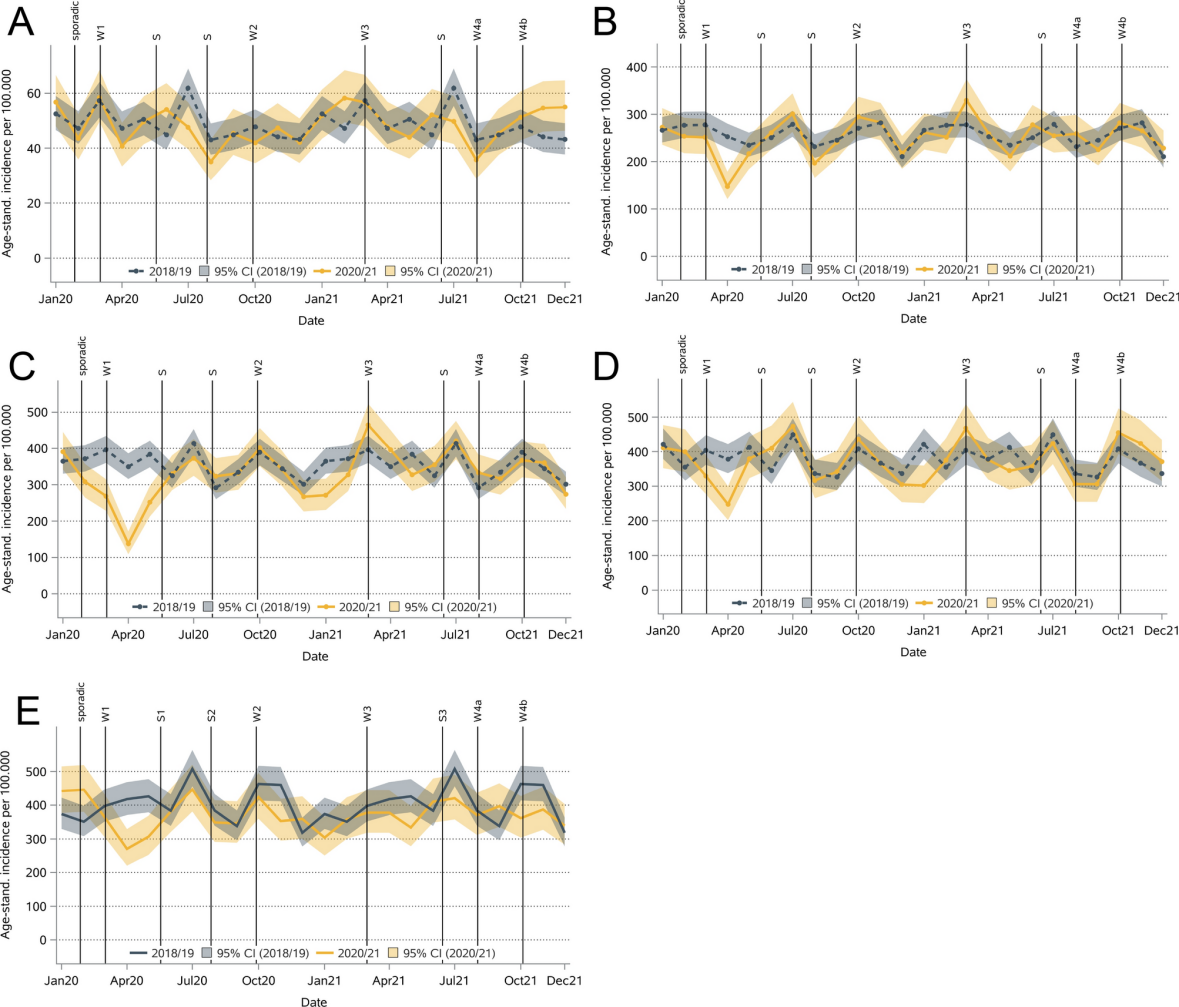
Monthly age-standardized incidence for invasive breast cancer by age at diagnosis (A: < 50, B: 50–59, C: 60–69, D: 70–79, E: 80 +).

**Fig. 4:**
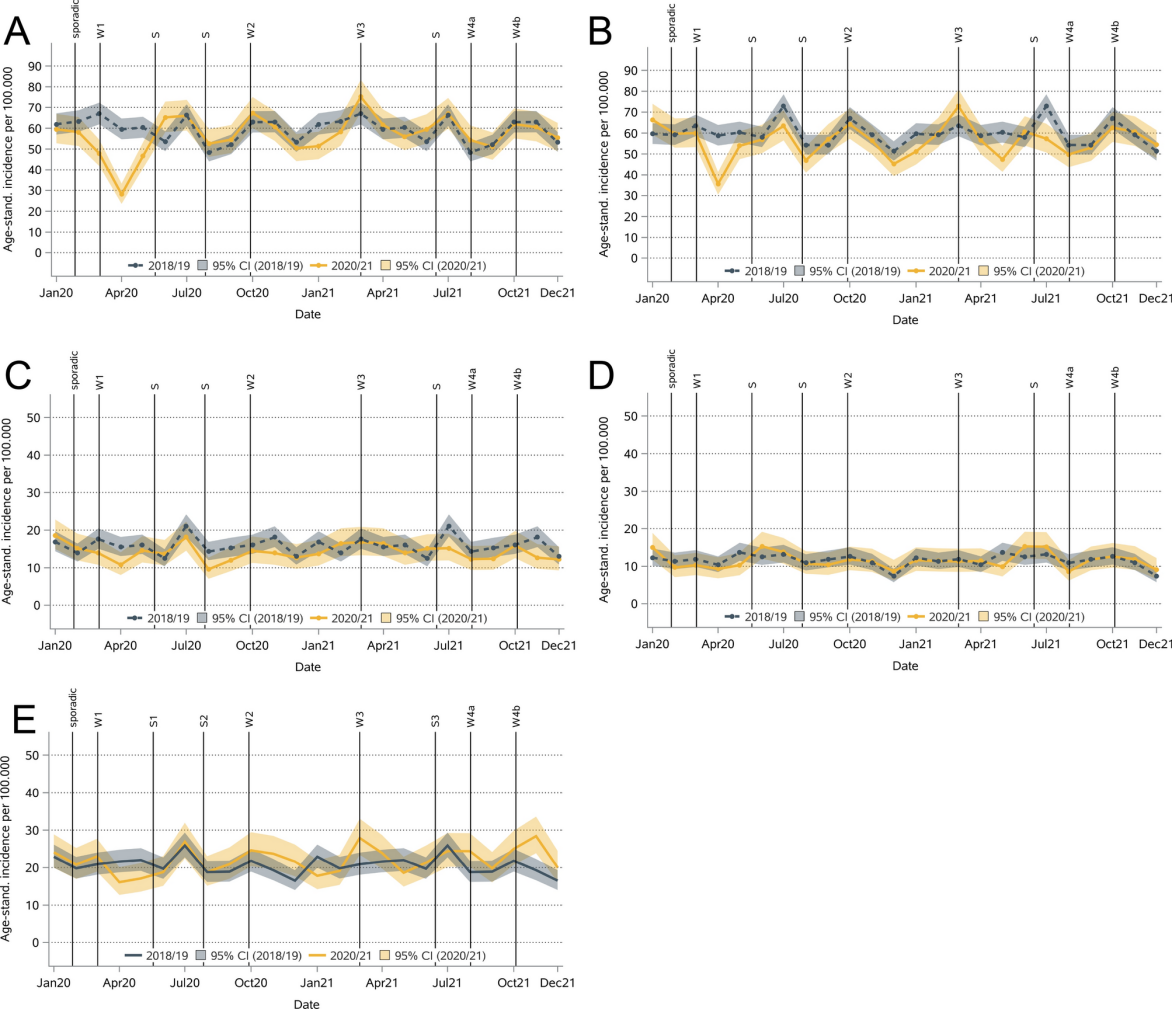
Monthly age-standardized incidence for breast cancer by stage at diagnosis (A: I, B: II, C: III, D: IV, E: Missing).

The decrease in invasive BC incidence in 2020 was significant in the age groups 60–69 (-13%) and 80 + years (-7%) and for stage I (-8%), stage II (-7%), and stage III (-12%, Table 2 & Figs. 3 and 4) patients. A decrease in April 2020 was detected in all these subgroups except for stage III. In 2021, the incidence was significantly lower only for the age group 80 + (-8%) and for stage II (-4%) and III (-9%). For non-invasive BC, the incidence decreased in all age groups in 2020, but the differences were significant only for those aged 50–59 years (-6%, Table 2). No significant increase or decrease was observed in 2021.

### Characteristics of early-stage BC patients

A total of 22,708 patients with early-stage BC were included (Table 3). The median age at diagnosis was 62 years. Most patients had ductal carcinoma (75%), intermediate grade (62%), stage IA (60%), HR + (89%; 88% ER + , 80% PGR +) and HER2- (88%) tumors. Compared to those in 2018/2019, the proportion of patients with PGR + tumors decreased significantly from 81 to 79% in 2020, while all other characteristics were comparable. Patients diagnosed in 2021 were less likely to be aged 70–79 years but more likely to be aged 60–69 years, more likely to have other/unspecified histology (6% vs. 4%), intermediate grade (65% vs. 61%), HR- (12% vs. 10%) and HER2- (89% vs. 88%) tumors.

**Table 3:** Patient and tumor characteristics of early-stage breast cancer patients by calendar period.

	Calendar year			Comparison to 2018/2019	
	2018/2019	2020	2021	*P* value 2020	*P* value 2021
N	11,579	5,380	5,749		
Age at diagnosis					
Median (IQR)	62 (20)	62 (20)	62 (18)		
0–49 years	1,942 (16.8%)	855 (15.9%)	922 (16.0%)	0.48	**0.02**
50–59 years	3,036 (26.2%)	1,440 (26.8%)	1,491 (25.9%)		
60–69 years	3,150 (27.2%)	1,456 (27.1%)	1,694 (29.5%)		
70–79 years	,2248 (19.4%)	1,037 (19.3%)	1,043 (18.1%)		
80 + years	1,203 (10.4%)	592 (11.0%)	599 (10.4%)		
Histology					
Ductal	8,737 (75.5%)	4,025 (74.8%)	4,261 (74.1%)	0.19	** < 0.001**
Lobular	1,707 (14.7%)	798 (14.8%)	825 (14.4%)		
Ductal & Lobular	72 (0.6%)	35 (0.7%)	31 (0.5%)		
Other, specified	649 (5.6%)	289 (5.4%)	296 (5.1%)		
Unspecified	414 (3.6%)	233 (4.3%)	336 (5.8%)		
Grade					
Low (G1)	1,623 (14.5%)	748 (14.6%)	716 (13.3%)	0.39	** < 0.0001**
Intermediate (G2)	6,824 (61.2%)	3,184 (62.1%)	3,480 (64.7%)		
High (G3)	2,708 (24.3%)	1,195 (23.3%)	1,183 (22.0%)		
Pre-surgical stage^*a*^					
0	487 (4.2%)	226 (4.2%)	241 (4.2%)	0.90	0.20
IA	6,922 (59.8%)	3,205 (59.6%)	3,512 (61.1%)		
IB	54 (0.5%)	21 (0.4%)	18 (0.3%)		
IIA	4,116 (35.5%)	1,928 (35.8%)	1,978 (34.4%)		
Hormone receptor					
Negative	1,173 (10.4%)	616 (11.5%)	680 (12%)	**0.03**	**0.002**
Positive	10,069 (89.6%)	4,720 (88.5%)	4,985 (88%)		
Estrogen receptor					
Negative	1,322 (11.8%)	673 (12.6%)	738 (13%)	0.12	**0.02**
Positive	9,910 (88.2%)	4,662 (87.4%)	4,930 (87%)		
Progesterone receptor					
Negative	2,132 (19%)	1,144 (21.5%)	1,213 (21.5%)	**0.0002**	**0.0001**
Positive	9,104 (81%)	4,185 (78.5%)	4,437 (78.5%)		
HER2 status					
Negative	9,607 (87.7%)	4,619 (88.8%)	4,931 (89.0%)	0.05	**0.02**
Positive	1,344 (12.3%)	582 (11.2%)	610 (11.0%)		

IQR = interquartile range, P value = P value from the chi-square test, significant values are shown in bold, HER2 = human epidermal growth factor receptor 2; ^a^ If the pre-surgical stage was not available, it was completed with staging information after surgery.

### Neoadjuvant therapy in patients with early-stage BC

Overall, administration of neoadjuvant therapy was slightly higher in 2021 (18.1%) compared to 2018/2019 (16.2%, Table 4). In 2020, it was slightly lower (15.5%). Administration decreased with age and stage, irrespective of the calendar year. Patients with preoperative stage IIA, negative HR status or positive HER2 status were more likely to receive neoadjuvant therapy. Regarding neoadjuvant endocrine therapy, for only 11,439 (57.9%) of the 19,744 HR + patients, an administration of endocrine therapy was notified to the cancer registry, indicating an underreporting in the registry dataset. The proportion decreased from 60.2% in 2018/2019 and 56.6% in 2020 to 54.2% in 2021. If endocrine therapy was administered, it was in a neoadjuvant setting in 637 (5.6%) patients. This proportion was particularly increased in 2021 (242 (9.0%)) compared to 107 (4.0%) in 2020 and 288 (4.8%) in 2018/2019.

**Table 4:** Number and proportion of patients with early-stage breast cancer with neoadjuvant therapy.

	Calendar year		
	2018/2019	2020	2021
Overall	1,878 (16.2%)	834 (15.5%)	1,039 (18.1%)
Age at diagnosis			
0–49 years	619 (31.9%)	300 (35.1%)	323 (35.0%)
50–59 years	530 (17.5%)	211 (14.7%)	307 (20.6%)
60–69 years	452 (14.3%)	191 (13.1%)	265 (15.6%)
70–79 years	252 (11.2%)	112 (10.8%)	118 (11.3%)
80 + years	25 (2.1%)	20 (3.4%)	26 (4.3%)
Grade			
Low (G1)	56 (3.5%)	16 (2.1%)	23 (3.2%)
Intermediate (G2)	647 (9.5%)	248 (7.8%)	415 (11.9%)
High (G3)	1,147 (42.4%)	534 (44.7%)	527 (44.5%)
Pre-surgical stage^*a*^			
0/I	837 (11.2%)	372 (10.8%)	465 (12.3%)
IIA	1,041 (25.3%)	462 (24.0%)	574 (29.0%)
Hormone receptor			
Negative	599 (51.1%)	323 (52.4%)	392 (57.6%)
Positive	1,234 (12.3%)	510 (10.8%)	629 (12.6%)
HER2 status			
Negative	1,199 (12.5%)	567 (12.3%)	727 (14.7%)
Positive	617 (45.9%)	259 (44.5%)	287 (47.0%)

^a^ If the pre-surgical stage was not available, it was completed with staging information after surgery.

### Time to surgery for early-stage BC patients

**Fig. 5:**
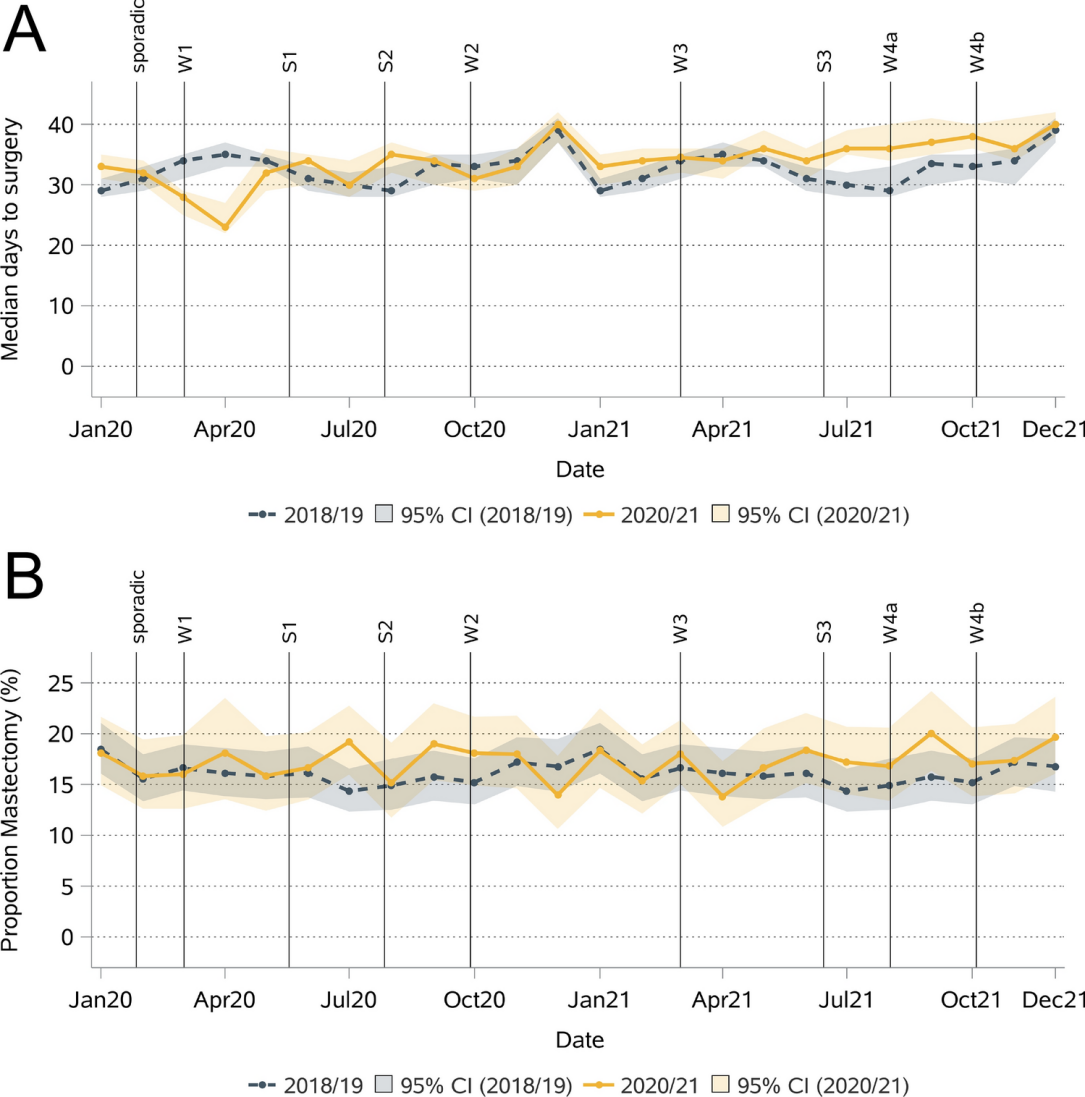
Median days to surgery (**A**) and mastectomy rate (**B**) for early-stage breast cancer per month.

The median (and mean) time to surgery was 33 (62) days in 2018/2019, 32 (60) days in 2020 and 36 (64) days in 2021 (Table 5). According to the adjusted models, the decrease in time to surgery from 2018/2019 to 2020 was statistically significant (IRR 0.95 (0.93–0.97)) and could not be explained by changes in the use of neoadjuvant therapy. The time to surgery was particularly short around April 2020 (Fig. 5). Subgroup analyses revealed a significantly shorter time to surgery in 2020 for patients aged 50–59 and 70–79 years, intermediate-grade tumors, pre-surgical stage IA and IIA, HR + , and HER2- tumors, and patients who received a BCS or did not receive neoadjuvant therapy. A significantly longer time to surgery in 2021 than in 2018/2019 was observed for patients aged 70 + years, those with intermediate-grade tumors, and those with HR- and HER2- tumors. The longer time could be explained by the administration of neoadjuvant therapy for all subgroups except patients aged 70 + years.H:\journals\Springer\SpACE\41598\75084\Stage200\Contents\LE

**Table 5:** Mean and median time to surgery in early-stage breast cancer patients.

	Mean (std)			Mean (std)			IRR (95% CI)^a^		IRR (95% CI)^b^	
	Calendar year			Calendar year			Reference: 2018/2019		Reference: 2018/2019	
	2018/19	2020	2021	2018/19	2020	2021	2020	2021	2020	2021
Overall	**62 (70)**	**60 (67)**	**64 (69)**	**33 (36)**	**32 (34)**	**36 (36)**	**0.95 (0.93–0.98)**	1.02 (1.00–1.05)	**0.95 (0.93–0.97)**	1.00 (0.98–1.02)
Age at diagnosis										
0–49 years	90 (89)	92 (86)	89 (85)	36 (168)	37 (168)	39 (164)	0.95 (0.89–1.02)	0.97 (0.91–1.04)	0.96 (0.92–1.02)	**0.93 (0.88–0.98)**
50–59 years	69 (74)	64 (70)	72 (73)	37 (43)	35 (35)	41 (43)	**0.93 (0.88–0.97)**	1.02 (0.97–1.07)	**0.93 (0.89–0.97)**	0.98 (0.94–1.02)
60–69 years	62 (68)	60 (64)	63 (66)	36 (35)	36 (32)	37 (30)	0.96 (0.91–1.00)	1.00 (0.96–1.05)	0.96 (0.92–1.00)	0.98 (0.94–1.02)
70–79 years	45 (54)	43 (53)	49 (58)	27 (23)	27 (23)	29 (22)	**0.96 (0.91–1.02)**	**1.09 (1.03–1.15)**	**0.93 (0.89–0.97)**	**1.07 (1.02–1.13)**
80 + years	30 (27)	33 (32)	37 (35)	25 (20)	26 (20)	29 (20)	0.95 (0.98–1.12)	**1.17 (1.10–1.25)**	1.01 (0.95–1.07)	**1.11 (1.04–1.18)**
Grade										
Low (G1)	34 (25)	33 (26)	35 (24)	29 (23)	28 (21)	30 (20)	0.96 (0.91–1.01)	1.05 (0.99–1.11)	0.96 (0.92–1.01)	1.04 (0.99–1.09)
Intermediate (G2)	47 (54)	44 (49)	49 (53)	30 (26)	29 (23)	34 (26)	**0.95 (0.92–0.98)**	**1.05 (1.02–1.08)**	**0.96 (0.93–0.98)**	1.02 (0.99–1.04)
High (G3)	115 (93)	113 (90)	113 (91)	73 (181)	67 (174)	64 (176)	0.97 (0.91–1.03)	0.98 (0.93–1.04)	0.92 (0.88–0.96)	0.95 (0.91–1.00)
Pre-surgical stage^*a*^										
0	45 (39)	50 (37)	58 (52)	37 (32)	41 (33)	41 (39)	1.15 (0.97–1.38)	1.08 (0.89–1.30)	1.02 (0.88–1.19)	1.04 (0.88–1.21)
IA	53 (61)	51 (59)	53 (58)	31 (28)	31 (26)	34 (27)	**0.97 (0.93–1.00)**	1.03 (0.99–1.05)	**0.96 (0.93–0.98)**	1.01 (0.98–1.03)
IB	32 (31)	37 (54)	25 (13)	26 (20)	21 (16)	22 (15)	0.98 (0.63–1.51)	0.82 (0.51–1.30)	0.89 (0.64–1.24)	1.07 (0.73–1.55)
IIA	81 (83)	75 (79)	85 (84)	36 (140)	34 (118)	40 (147)	**0.93 (0.89–0.97)**	1.04 (0.99–1.09)	**0.93 (0.90–0.97)**	0.99 (0.96–1.03)
Hormone receptor										
Negativ	133 (92)	135 (89)	149 (87)	164 (181)	170 (176)	184 (172)	1.04 (0.96–1.12)	**1.09 (1.01–1.18)**	0.98 (0.93–1.05)	1.03 (0.97–1.10)
Positiv	54 (63)	50 (57)	53 (58)	31 (30)	30 (27)	34 (27)	**0.95 (0.92–0.97)**	1.02 (0.99–1.04)	**0.95 (0.92–0.97)**	0.99 (0.98–1.02)
HER2 Status										
Negativ	54 (64)	53 (62)	57 (63)	30 (29)	30 (28)	35 (29)	**0.96 (0.93–0.98)**	**1.03 (1.00–1.05)**	**0.96 (0.94–0.98)**	1.00 (0.98–1.02)
Positiv	124 (90)	117 (87)	124 (88)	139 (176)	119 (172)	148 (163)	0.96 (0.88–1.04)	1.02 (0.94–1.11)	0.91 (0.85–0.97)	0.98 (0.93–1.05)
Type of surgery										
Breast-conserving	61 (69)	57 (66)	61 (67)	33 (35)	35 (33)	35 (33)	**0.94 (0.91–0.96)**	1.00 (0.98–1.03)	**0.95 (0.93–0.97)**	1.00 (0.98–1.02)
Mastectomy	71 (76)	71 (74)	77 (76)	35 (54)	40 (56)	40 (56)	0.97 (0.92–1.03)	1.04 (0.98–1.10)	0.96 (0.92–1.01)	0.99 (0.94–1.04)
Neoadj. therapy										
No	38 (39)	36 (34)	40 (37)	29 (23)	29 (21)	31 (22)	**0.94 (0.92–0.96)**	1.01 (0.99–1.04)	/	/
Yes	186 (67)	188 (59)	176 (72)	203 (61)	200 (53)	196 (71)	1.01 (0.97–1.05)	**0.95 (0.91–0.98)**	/	/

Std = standard deviation, IQR = interquartile rage, IRR = incidence rate ratio, CI = confidence interval, Neoadj. = neoadjuvant; ^a^ incidence rate ratios were extracted from a negative binomial regression model with adjustment for age, grade, pre-surgical stage, hormone receptor status, and HER2 status (stratification factor was omitted from the model).  ^b^ incidence rate ratios with additional adjustment for neoadjuvant therapy. Significant values are shown in bold.

### Type of surgical treatment in early-stage BC patients

The proportion of patients with early-stage BC who underwent mastectomy increased from 16% in 2018/2019 to 17% in 2020 and 2021 (Table 6). Monthly analyses indicated a greater proportion of mastectomies in the summer of 2020 and 2021 than in the summer of the reference years, but this interpretation is hampered by the large random variation (Fig. 5). After adjustment for differences in patient and tumor characteristics, the odds of mastectomy were 13% greater in 2021 than in 2018/2019. Stratified analyses revealed the strongest differences for high-grade tumors, with 287% greater odds of mastectomy in 2020 and 2021 than in 2018/2019. Significantly greater odds of mastectomy in 2021 than in 2018/2019 were also observed for patients aged 50–59 years, with intermediate-grade, pre-surgical stage 0/I, HR + and HER2- tumors, and with neoadjuvant therapy.

**Table 6:** Number and proportion of patients with early-stage breast cancer with mastectomy (instead of breast-conversing surgery).

	Calendar year			Comparison to 2018/2019	
	2018/2019	2020	2021	OR (95% CI)^a^ 2020	OR (95% CI)^a^ 2021
Overall	1,862 (16.1%)	921 (17.1%)	997 (17.3%)	1.08 (0.98–1.19)	**1.13 (1.03–1.24)**
Age at diagnosis					
0–49 years	448 (23.1%)	226 (26.4%)	252 (27.3%)	1.16 (0.95–1.41)	1.19 (0.98–1.44)
50–59 years	314 (10.3%)	163 (11.3%)	183 (12.3%)	1.19 (0.97–1.47)	**1.34 (1.09–1.64)**
60–69 years	299 (9.5%)	144 (9.9%)	154 (9.1%)	1.04 (0.84–1.30)	0.97 (0.78–1.20)
70–79 years	403 (17.9%)	193 (18.6%)	198 (19.0%)	1.04 (0.86–1.28)	1.19 (0.97–1.45)
80 + years	398 (33.1%)	195 (32.9%)	210 (35.1%)	0.93 (0.75–1.17)	1.01 (0.81–1.26)
Grade					
Low (G1)	138 (8.5%)	58 (7.8%)	47 (6.6%)	0.95 (0.68–1.33)	0.73 (0.51–1.05)
Intermediate (G2)	1,138 (16.7%)	545 (17.1%)	618 (17.8%)	1.02 (0.91–1.15)	**1.14 (1.01–1.28)**
High (G3)	524 (19.4%)	280 (23.4%)	278 (23.5%)	**1.27 (1.07–1.51)**	**1.27 (1.07–1.51)**
Pre-surgical stage^*b*^					
0/I	796 (10.7%)	387 (11.2%)	438 (11.6%)	1.09 (0.95–1.25)	**1.15 (1.01–1.32)**
IIA	1,066 (25.9%)	534 (27.7%)	559 (28.3%)	1.06 (0.93–1.20)	1.11 (0.98–1.26)
Hormone receptor					
Negative	280 (23.9%)	145 (23.5%)	172 (25.3%)	1.02 (0.79–1.30)	1.13 (0.89–1.43)
Positive	1,520 (15.1%)	769 (16.3%)	810 (16.2%)	1.09 (0.99–1.21)	**1.13 (1.03–1.25)**
HER2 Status					
Negative	1,460 (15.3%)	758 (16.4%)	814 (16.5%)	1.07 (0.97–1.19)	**1.14 (1.03–1.26)**
Positive	288 (21.4%)	135 (23.2%)	140 (23.0%)	1.13 (0.88–1.44)	1.11 (0.87–1.42)
Neoadjuvant therapy					
No / not reported	1,496 (15.4%)	722 (15.9%)	746 (15.8%)	1.04 (0.94–1.16)	1.09 (0.98–1.21)
Yes	366 (19.5%)	199 (23.9%)	251 (24.2%)	1.20 (0.97–1.48)	**1.29 (1.06–1.57)**

OR = odds ratio, CI = confidence interval; significant values are shown in bold; ^a^ odds ratios were extracted from a logistic regression model adjusted for age, grade, pre-surgical stage, hormone receptor status, HER2 status, and neoadjuvant therapy (the stratification factor was omitted from the model). ^b^ If the pre-surgical stage was not available, it was completed with staging information after surgery.

### Adjuvant radiotherapy in early-stage BC patients

Overall, 62.4% of patients with early-stage BC received adjuvant radiotherapy, with 71.9% receiving it after BET and 15.0% after MAS. The rate of adjuvant radiotherapy were lower in 2021 than in 2018/2019, overall and stratified by type of surgery (Table 7). After adjustment, the decrease was significant overall, for intermediate- and high-grade tumors, and after BET. In 2020, rates were only significantly lower for patients with high-grade tumors.

**Table 7:** Number and proportion of patients with early-stage breast cancer with adjuvant radiotherapy.

	Calendar year			Comparison to 2018/2019	
	2018/2019	2020	2021	OR (95% CI)^a^ 2020	OR (95% CI)^a^ 2021
Overall	7,335 (63.3%)	3,351 (62.3%)	3,491 (60.7%)	0.94 (0.88–1.01)	**0.85 (0.79–0.91)**
Grade					
Low (G1)	1,231 (74.7%)	565 (75.5%)	522 (72.9%)	1.00 (0.81–1.24)	0.87 (0.71–1.08)
Intermediate (G2)	4,332 (63.5%)	1,999 (62.8%)	2,124 (61.0%)	0.97 (0.88–1.06)	**0.84 (0.77–0.92)**
High (G3)	1,540 (56.9%)	638 (53.4%)	647 (54.7%)	**0.83 (0.72–0.96)**	**0.86 (0.74–1.00)**
Type of surgery					
Breast-conserving	7,048 (72.5%)	3,213 (72.1%)	3,351 (70.5%)	0.96 (0.88–1.05)	**0.87 (0.80–0.94)**
Mastectomy	287 (15.4%)	138 (15.0%)	140 (14.0%)	0.94 (0.74–1.19)	0.84 (0.66–1.07)

OR = odds ratio, CI = confidence interval; significant values are shown in bold; ^a^ odds ratios were extracted from ^a^ logistic regression model adjusted for age, grade, pre-surgical stage, hormone receptor status, HER2 status, and neoadjuvant treatment.

## Discussion

The age-standardized incidence of invasive and non-invasive BC decreased during the pandemic in April 2020 but recovered quickly, resulting in comparable incidences in 2021 and 2018/2019. However, women aged 80 years or older still had a decreased incidence of invasive BC in 2021. The time to surgery was slightly shorter in 2020 but longer in patients aged 70 and older in 2021. The proportion of patients who underwent mastectomy increased overall in 2021. For patients with high-grade tumors, it was already increased in 2020.

In Germany, women aged 50 to 69 years are invited to the organized mammography screening program every two years. On 25 March 2020, the Joint Federal Committee (G-BA) decided to suspend the invitation system for the mammography screening program until 30 April 2020^6^. There was no clear regulation for already allocated mammography screening appointments, although in most parts of Germany, screening was interrupted in April for reasons of infection protection and women were actively uninvited. Thus, the observed decline cancer incidence in April 2020 was to be expected. While the screening participation rates in 2018/2019 (50%) and 2020 (49%) were almost comparable, the invitation rate decreased from 95% in 2018/2019 to 90% in 2020^6^. After the pause of the program, screening units extended their opening hours and made up for more than half of the missed screenings in 2020. Health claims data analyses revealed that 11% fewer mammography screens were conducted in 2020 than in 2017–2019^12^. In 2021, the screening invitation (97%) and participation rate (51%) were slightly above average^13^. A fast return to normal incidence rates was also stipulated by press releases from the German Cancer Research Center (DKFZ), the German Cancer Aid, and the German Cancer Society in April 2020^14^. The task force advised the public to attend appointments for examinations to clarify suspicious symptoms as soon as possible to prevent late-stage cancers. Consequently, despite the ongoing threat of the COVID-19 pandemic in 2020 and 2021, further lockdowns, and recommendations to avoid contact, the age-standardized incidence already reached the levels of the reference period during the summer of 2020. In 2021, BC incidence was reduced only in women aged 80 years and older who were also at increased risk of a severe course of COVID-19 infection. The results for all of Germany are only available for 2020 and show a reduction in the BC incidence rate of approximately 5% in 2020 compared to 2019^2^. Other countries also suspended their mammography screening programs, mostly between the end of March and the middle to the end of May^15^. The temporal trends of BC incidence in other countries showed similar patterns. However, the magnitude of the reduction and the time to recovery to pre-pandemic levels vary strongly. For example, the incidence of BC declined by 5% in Denmark^16^, 7% in Madrid (Spain)^17^, 8% in Belgium^18^, and 19% in the United Kingdom in 2020^19^. The incidence estimates recovered to pre-pandemic levels in Denmark in the first half of 2021 and in the Netherlands between May and December 2021^16^. A higher incidence in 2021 than in the reference years was reported for Madrid (Spain, + 14%)^17^, and Norway (+ 10% in May to December)^20^. While the fast recovery to pre-pandemic levels is promising, a catch-up of cases missed during the pandemic phase was observed only in some countries by the end of 2021. In Baden–Württemberg, 7% of potentially missed cases in 2020 were not caught-up in 2021 indicating that women were still reluctant to go to mammography screening and/or clarify symptoms. Incidence rates and stage distributions should be monitored in the upcoming years to evaluate whether the pandemic led to a stage shift at time of diagnosis. Furthermore, some of the missed diagnoses may have never occurred because the affected individual died from competing causes (including COVID-19 infection) before the cancer occurred or was detected. This is a likely scenario as the COVID-19 pandemic caused deaths primarily in individuals who also had an elevated cancer risk, such as the elderly, smokers, obese individuals, or people with diabetes.

The pandemic not only influenced cancer diagnosis but also cancer care. Adapted treatment recommendations were rapidly developed^7^. Based on expert opinions, primary surgery of low-risk early-stage BC could safely be delayed for up to 12 weeks. Furthermore, for luminal-like BC, neoadjuvant/preoperative endocrine treatment was suggested for selected patients to avoid harm due to the delay of surgery. However, our results revealed a shorter time to surgery in 2020 than in the reference period, especially in April 2020. Reduced case numbers and the successful prioritization of invasive BC surgery during this time can probably explain this result. Similar results were reported from the Netherlands for stage I and II BC patients diagnosed during the lockdown and care restart^21^. In contrast, a study from the US reported a greater proportion of delayed surgery (> 90 days between diagnosis and surgery) in BC patients diagnosed in 2020 (9.6%) than in those diagnosed during the reference period (8.0%)^22^. In 2021, we found an increase in the time to surgery for the subgroups of patients aged 70 + , intermediate-grade, HR-, and HER2- BCs. However, this increase could be explained by administration of neoadjuvant therapy in all subgroups except patients aged 70 + . A multinational study showed that treatment delays during the pandemic varied among countries^23^. Delaying BC surgery can lead to upstaging, worse cancer prognosis^24,25^, and psychological distress^26^. Thus, the longer time to surgery in 2021, especially in elderly patients, necessitates monitoring the time to surgery in the following years.

While no increase in time to surgery was observed for HR + patients, our results showed an increased utilization of neoadjuvant endocrine therapy in 2021. Data quality was not sufficient to conduct detailed analyses and results should be interpreted with caution. However, the trend is in alignment with the expert opinion during the pandemic described above and necessitates further monitoring and evaluation of its impact on BC outcomes.

BCS is recommended for early-stage BC. This recommendation did not change during the pandemic. However, anecdotal evidence from patients’ reports at the German Cancer Information Service has shown that physicians may have recommended mastectomy to avoid adjuvant radiotherapy. Our results support this finding, as the odds of mastectomy versus BCS increased for high-grade tumors (+ 28%) in 2020 and overall (+ 13%) in 2021. In alignment, the odds of adjuvant radiotherapy were decreased for high-grade tumors (-17%) in 2020 and overall (-15%) in 2021. However, no clear temporal trends for mastectomy rates by month of diagnosis were observed, except for a slight tendency toward more mastectomy during the summer months. The results from the Netherlands show a significant increase in the odds of mastectomy for stage II patients (+ 21%) and a similar tendency for stage I patients (+ 23%) during care restart^21^. These associations were not present after restricting to nonscreening-detected tumors. Data from the US revealed a greater proportion of BC patients who underwent mastectomy in 2020 than in 2019 (+ 10%), but the analyses were neither restricted to early-stage BC nor adjusted for stage^27^. BCS plus radiotherapy is prognostically equivalent to mastectomy but is associated with a better body image and better long-term quality of life^28^. Although the increase in mastectomy for high-grade early BC in 2020 could be explained by the uncertainty regarding the risk of the virus during this time, the elevated mastectomy rate in 2021 is not plausible, as adapted cancer treatment guidelines were already developed, vaccination had started, and recommendations for dealing with the pandemic were in place.

The strengths of this first cancer registry-based study of the impact of the pandemic on BC incidence and surgical treatment in southern Germany are its population-based design (covering 11.07 million inhabitants) and the resulting large number of BC patients. Furthermore, the data set allowed monthly analyses and the use of pre-surgical staging information. The lack of population data by month is a limitation that requires the assumption of a constant population size throughout the year. Approximately 11% of the patients had to be excluded because no surgery or procedure code was reported to the cancer registry. While a minority of patients may not have received surgery, this proportion partly reflects underreporting of treatment. However, there was no temporal trend in the proportion of patients without notification of surgery. Thus, this limitation should not affect the comparison between the pandemic and the reference period. Data quality for neoadjuvant therapy and adjuvant radiotherapy was limited and showed a potential underreporting, particularly for endocrine therapy. Therefore, detailed analyses were not possible and results should be interpreted with caution. Due to the same reason, information on other factors routinely collected in the registry, e.g. other treatments and tumor size, could not be used in the analyses. Information on mammographic tumor size might have been a better predictor for mastectomy than staging. However, this information was not available.

## Conclusions

Our study showed that the age-standardized incidence of invasive and non-invasive BC decreased early during the COVID-19 pandemic but recovered by summer 2020. However, there was no catch-up of missed BC patients until the end of 2021. Furthermore, for women aged 80 + years, incidence was still 8% lower in 2021. Whether the missed cases will lead to a stage shift in the upcoming years needs to be monitored. We did not observe a strong impact on the time to surgery for early-stage BC patients. A tendency toward a greater proportion of mastectomies instead of BCS was observed during the pandemic years, although a change in the type of surgery was not recommended. This result underlines the importance of good communication and guidelines to find the best possible cancer treatment in such uncertain times.

## Data Availability

The data that support the findings of this study are available from the Baden-Württemberg Cancer Registry but restrictions apply to the availability of these data, which were used under license for the current study, and so are not publicly available. Data are however available from the authors upon reasonable request and with permission of the Baden-Württemberg Cancer Registry (contact: l.jansen@dkfz-heidelberg.de).

## Abbreviations

BC: Breast cancer
BCS: Breast-conserving surgery
ICD: International classification of diseases
UICC: Union for international cancer control
ER: Estrogen receptor
PR: Progesterone receptor
HER2: Human epidermal growth factor receptor 2; hormone receptor status
